# Faces with Light Makeup Are Better Recognized than Faces with Heavy Makeup

**DOI:** 10.3389/fpsyg.2016.00226

**Published:** 2016-03-01

**Authors:** Keiko Tagai, Hitomi Ohtaka, Hiroshi Nittono

**Affiliations:** ^1^Shiseido Global Innovation Center, Shiseido Company, LimitedKanagawa, Japan; ^2^Graduate School of Integrated Arts and Sciences, Hiroshima UniversityHiroshima, Japan

**Keywords:** female faces, makeup, memory, attractiveness, impression

## Abstract

Many women wear facial makeup to accentuate their appeal and attractiveness. Makeup may vary from natural (light) to glamorous (heavy), depending of the context of interpersonal situations, an emphasis on femininity, and current societal makeup trends. This study examined how light makeup and heavy makeup influenced attractiveness ratings and facial recognition. In a rating task, 38 Japanese women assigned attractiveness ratings to 36 Japanese female faces with no makeup, light makeup, and heavy makeup (12 each). In a subsequent recognition task, the participants were presented with 36 old and 36 new faces. Results indicated that attractiveness was rated highest for the light makeup faces and lowest for the no makeup faces. In contrast, recognition performance was higher for the no makeup and light make up faces than for the heavy makeup faces. Faces with heavy makeup produced a higher rate of false recognition than did other faces, possibly because heavy makeup creates an impression of the style of makeup itself, rather than the individual wearing the makeup. The present study suggests that light makeup is preferable to heavy makeup in that light makeup does not interfere with individual recognition and gives beholders positive impressions.

## Introduction

Many women wear facial makeup in their daily lives to accentuate their attractiveness and create favorable impressions. However, what type of makeup currently creates the most appealing and memorable impressions is not always evident. If people were aware of the social and psychological effects of makeup on the beholder, they would be more satisfied with, and knowledgeable about, their selection and use of cosmetic products.

The positive bias toward attractive individuals is known as the beauty halo effect or a beauty-is-good stereotype (Zebrowitz, 1997; Etcoff, 1999; Lemay et al., 2010; Lorenzo et al., 2010; Etcoff et al., 2011). For example, attractive people are assumed to have better personalities, greater abilities, and higher moral standards when compared with unattractive people. Makeup is expected to improve facial attractiveness and affect interpersonal perception.

However, the type of makeup that is considered most attractive to others is an unsettled issue. People’s positive attitudes toward the attractiveness of another’s makeup are affected by various factors, including societal and interpersonal contexts. For example, light makeup that enhances natural facial expressions is often considered attractive or appropriate in daily situations, whereas glamorous heavy makeup, which has greater contrasts as well as obvious coordination of the makeup applied to the eyes or lips with clothing or hairstyle, is considered more appropriate at events such as parties or celebrations. A previous study suggests that color cosmetics can be considered phenotypic extensions that are linked to biological meanings (Etcoff et al., 2011). Moreover, the luminance contrast in the face and redness of lips typically enhance attractiveness and femininity (Russell, 2003, 2009; Stephen and McKeegan, 2010). This effect is assumed to be due to the association of luminance and lip color with greater oxygenated blood perfusion, a state that typically reflects higher levels of estrogen levels, sexual arousal, and cardiac and respiratory health (Stephen and McKeegan, 2010).

Trends in beauty change over the course of time. The transition of fashion would be the transition of makeup because the trends in makeup and fashion are mutually interrelated (Blackman, 2012). Hunt et al. (2011) describes the history of cosmetics and beauty in the West. These trends are often affected by social background and public consciousness. For example, in Japan, after the enactment of *the Act on Securing, Etc. of Equal Opportunity and Treatment between Men and Women in Employment* in 1986, many working women have assumed roles in public life that are comparable to those of business men. The makeup trends at that time leaned toward heavy eyebrows and strongly accented facial features. Thus, working women during that period tended to wear heavy makeup. More recent trends in makeup have appeared since the Great East Japan Earthquake in 2011; these reveal a shift toward natural, lighter makeup. One interpretation of this trend is that lighter makeup expresses femininity or beauty in the search for healing during a time of confusion. Specific features of this trend can be seen in the increased brightness of the preferred colors for eyebrow accents and lip gloss; moreover, there is a growing emphasis on the expression of one’s personality as an element of natural femininity (Suzuki, 2015). Although there are some cultural differences, Cunningham et al. (1995) reported that the consistency of physical attractiveness ratings across ethnic groups was generally high. This is reasonable because attractiveness is not just a fashion but also has biological meanings (Etcoff, 1999).

Although makeup is applied with the goal of enhancing one’s attractiveness, whether a face with makeup is more or less memorable than one without makeup is unclear. Some studies suggest that attractive faces are more memorable. For instance, Marzi and Viggiano (2010) showed that recognition accuracy was higher for attractive faces than for unattractive faces and that the retrieval time for attractive faces was shorter than that for unattractive faces. In an event-related functional magnetic resonance imaging (fMRI) study, Tsukiura and Cabeza (2011) showed a higher level of functional connectivity between the orbitofrontal and hippocampal regions during the encoding of attractive faces than for unattractive faces. They suggested that attractive faces were more memorable than unattractive faces because reward-related activity in the orbitofrontal cortex enhanced encoding-related activity in the hippocampus. Another fMRI study showed that activities in the orbitofrontal cortex and the hippocampus were associated with the increased attractiveness of a female face with makeup compared with the same female face without makeup (Ueno et al., 2014). These findings suggest that memory processes interact with facial attractiveness.

On the other hand, the distinctiveness of the face is known to be a strong predictor of recognition. Because attractive faces exhibit greater similarity to each other, attractive faces are typical or ordinary, meaning that they should be less distinctive and harder to recognize (Light et al., 1981). Faces judged to be attractive tend to be related to facial averageness or proximity to the mathematical mean of faces in a population (Thornhill and Gangestad, 1993, 1999; Langlois et al., 1994; Fink and Penton-Voak, 2002). Recently, Wiese et al. (2014) reported that recognition accuracy was higher for unattractive than for attractive faces, even when faces were matched for distinctiveness on the basis of a rating. Based on the early posterior negativity (EPN) recorded in the learning phase, they argued that the processing of emotionally relevant attractive faces might hamper their encoding into memory. In contrast, other studies report no direct relationship between attractiveness and recognition memory (Cutler and Penrod, 1989; Brigham, 1990; Sarno and Alley, 1997). For example, Wickham and Morris (2003) showed that attractiveness had a negative correlation with measures of traditional distinctiveness (e.g., ease of spotting the face in a crowd) and deviational distinctiveness (e.g., deviation from an average face); however, attractiveness did not predict recognition memory.

Regarding the effect of makeup on facial recognition, Ueda and Koyama (2010) used a very short (150 ms) retention period and suggested the superiority of light makeup over heavy makeup. Participants were asked to judge whether two faces were the same person when two types of faces were presented in sequence: the first face for 300 ms, followed by a visual mask for 150 ms, and then the second face presented until a response was made. Results showed that light makeup made the recognition of a face easier than heavy makeup and no makeup.

**FIGURE 1:**
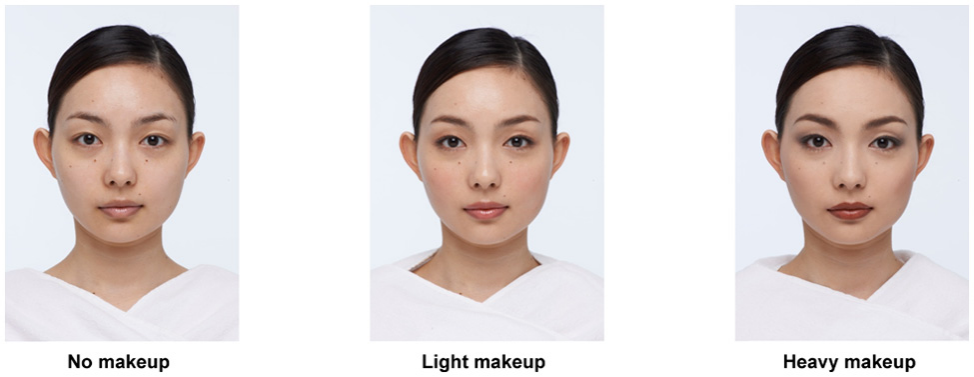
**Examples of facial images (no makeup, light makeup, and heavy makeup)**. For illustrative purpose, this figure shows the no makeup, light makeup, and heavy makeup versions of the same model. In the rating task (encoding phase), each participant viewed 36 models only once in one of the three versions (12 models each). The individual in this Figure has been informed consent to publish her face images.

In the present study, we examined the influence of light and heavy makeup on ratings of attractiveness and face recognition in a recognition task with a longer (i.e., a few minutes) retention period. Here we define the light makeup is a kind of makeup that is characterized by naturalness and femininity. Reddish colors were used and blended naturally into the skin. On the other hand, we define the heavy makeup as a kind of makeup that is characterized by perfectness, maturity, and coolness. Dark, low-chromatic colors were used to enhance the luminance contrast in the face. **Figure 1** shows examples of the three types of facial image. Given currently popular trends, we hypothesized that light makeup would obtain better attractiveness ratings than faces with no makeup or heavy makeup. If attractive faces have a direct relationship with face recognition, then light makeup should be remembered better. In contrast, if the distinctiveness of a face predicts subsequent memory performance, recognition accuracy should be highest for no makeup, middle for light makeup, and lowest for heavy makeup, because the heavier the makeup is, the less evident individual facial features are.

## Materials and Methods

### Participants

A total of 40 adult women were recruited by a research firm and paid for participation. The conditions of recruiting were (1) physically and mentally unimpaired, (2) being right-handed, (3) having reported normal or corrected to normal vision, (4) wearing makeup on a daily basis, and (5) being familiar with the effect of makeup on the impression of a face. Participants were gave written informed consent before testing. The Ethical Committee of Shiseido Global Innovation Center approved this study. Due to a technical failure in the experiment, the data of two participants could not be used. The following analysis was conducted on the data of 38 women (mean age: 27.1 ± 4.8 years).

### Stimuli

A total of 72 female face images with neutral facial expressions were used. Among them, 68 faces were of different female models (25–35 years old, taken by a professional photographer) and four faces were generated by using computer graphics. Even when these virtual faces were excluded from the analysis, the conclusion did not change. Therefore, the results of the whole dataset are reported here. There were three categories of faces: no makeup, light makeup, and heavy makeup. These faces were of different individuals. For the light makeup, foundation was blended smoothly into the skin tone. The outline of the facial features was blurred and the shape of the eyebrows was smoothed to create softness. The eye shadow was also blurred to blend into the skin tone. The lips were tinged with a reddish gloss. A soft red was used on the cheeks and blended from the center into the skin at the periphery. For the heavy makeup, a matte foundation covered the skin. The outline of the facial features was emphasized with straight lines. The eyebrows were drawn in a dark color with sharp, straight lines to make the center of the brow higher. Dark eye shadow was applied to produce long, slitted eyes. A matte brownish color with a sharp outline was added to the lips. Cheek color was a dark red–brown and blurred so as to accent the cheekbone. **Figure 1** shows examples of facial images. The quality of all the pictures was checked carefully by a professional makeup artist and a makeup product manager. When necessary, the pictures (including no makeup faces) were retouched by computer graphics to meet a standard of cosmetic quality and naturalness. The size of each picture was 500 × 368 pixels. A constant-size oval occluding window was applied to each photograph to hide the background and hair. The 72 facial images were divided in two sets of 36 faces (12 for each type).

### Procedure

The stimuli were presented on a computer screen (VIEWPixx, VPixx Technologies Inc.) in a dimly lit room by Inquisit 4.0 (Millisecond software). All the faces were displayed against a black background and subtended 8° wide × 10° height at a viewing distance of 70 cm.

**FIGURE 2:**
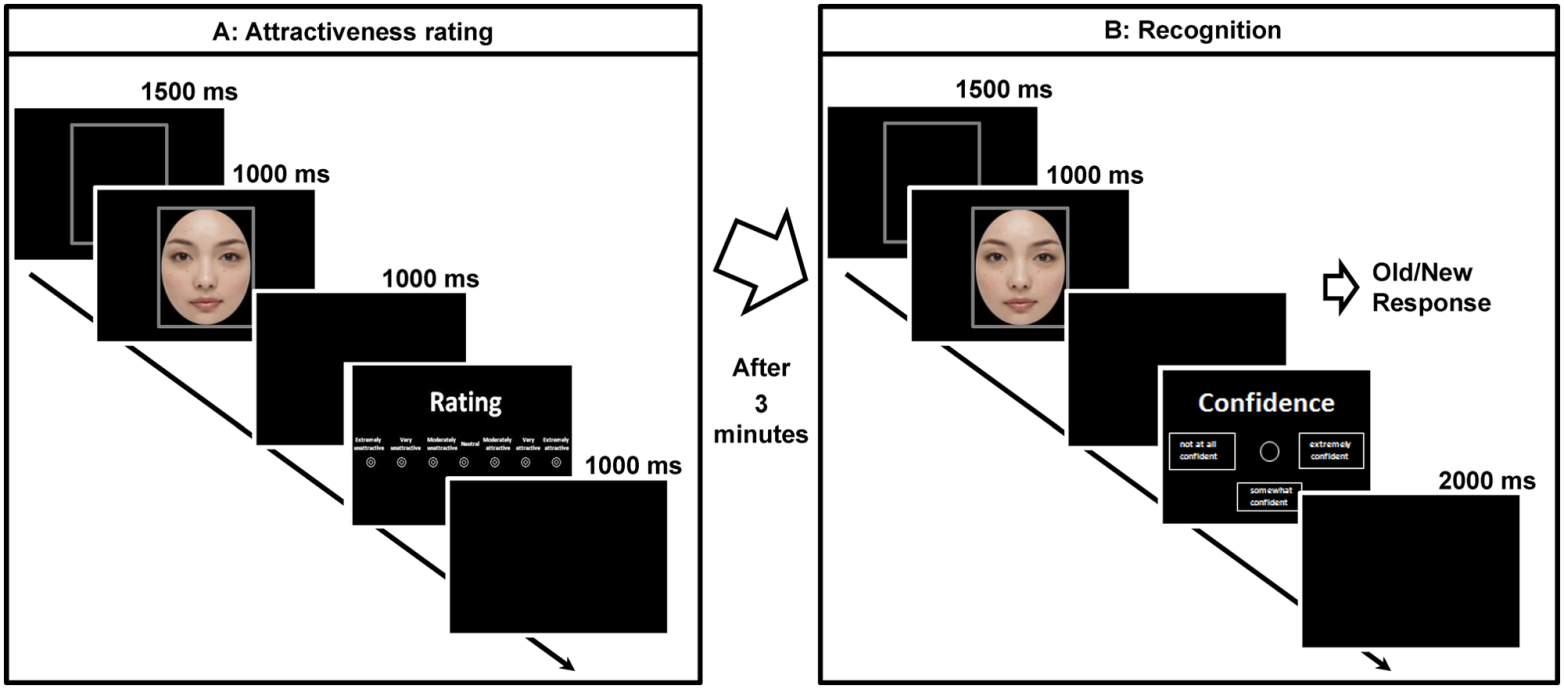
**Task design**. An attractiveness rating task **(A)** was followed by an incidental recognition task **(B)**.

**Figure 2** shows a schematic diagram of the experiment. The experiment consisted of two tasks. In a rating task, 36 images consisting of 12 no makeup, 12 light makeup, and 12 heavy makeup faces were presented one at a time. Each image appeared only once. After viewing a frame for 1500 ms, a face was displayed for 1000 ms. Subsequently, a blank frame appeared for 1000 ms, and then the rating screen was presented. The participants were instructed to rate each face for attractiveness by moving and clicking a computer mouse using a 7-point scale as follows: 1 = *extremely unattractive*, 2 = *very unattractive*, 3 = *moderately unattractive*, 4 = *neither*, 5 = *moderately attractive*, 6 = *very attractive*, 7 = *extremely attractive*. The next trial began 1000 ms after the response. The stimuli were presented in randomized order across participants.

The recognition task started with an interval of 3 minutes after the end of the rating phase. No advance notice was given for the recognition task. All the 72 images were presented one by one in randomized order; half were previously viewed images (“old”) and the other half were unviewed images (“new”). After viewing a frame for 1500 ms, a face was displayed for 1000 ms. The participants were asked to indicate whether they recognized the face image by choosing either the “old” or “new” button on a response pad (Cedrus RB-530). The response hands were counterbalanced across participants. After a button press, a confidence rating screen appeared on which participants rated their recognition confidence using a 3-point scale (1 = *not at all confident*, 2 = *somewhat confident*, and 3 = *extremely confident*) by button press. The next trial began 2000 ms after the confidence rating. The response time was not limited in either task. Before each task, the participants performed a practice trial with male facial images to ensure they were able to perform the task.

At the end of the experiment, participants were presented again with 36 facial images that were shown in the rating task and asked to choose any number of faces that they felt made an impression. Then they described the reasons for each choice on a piece of paper.

### Data Analysis

For the rating task, the mean subjective ratings of attractiveness of faces were analyzed. For the recognition task, hit (correct recognition) rates, correct rejection rates, and confidence ratings were analyzed. In addition, the geometric means of correct response times (i.e., from the onset of each stimulus to the onset of a response) were calculated. A receiver operating characteristic (ROC) curve was plotted for each makeup category and each participant. Each curve consisted of five points, which were calculated from five response criteria determined by a combination of the yes/no answers and confidence scores. Specifically, a “yes” answer with a high confidence score of three was regarded as the “yes” response at the highest response criterion, and a “yes” answer with a high or middle confidence score was regarded as the “yes” response at the second highest response criterion, and so on. Correct and false recognition rates were calculated for these five response criteria. The area under the ROC curve (AUC) was calculated as a measure of the sensitivity of face recognition. According to the previous study, the measures of discrimination accuracy (*Pr* = Hit – False recognition) and response bias [*Br* = False recognition/(1 – *Pr*)] were also calculated (Corwin, 1994). All the measures were analyzed by a repeated-measures multivariate analysis of variance (MANOVA) with a single factor: makeup (no makeup, light makeup, and heavy makeup). *Post hoc* pair-wise comparisons between means were made by *t*-tests with the Bonferroni correction. The alpha level was set to 0.05 for all analyses.

## Results

**Table 1** summarizes the mean values of 10 dependent measures for the three makeup categories, along with the statistical results. For the rating task, attractiveness differed significantly between the three makeup categories: light makeup received the highest ratings, heavy makeup was in second place, and no makeup received the lowest ratings.

**Table 1:** Summary of the subjective and behavioral results.

		Condition								
		No makeup		Light makeup		Heavy makeup		*F(2*,36)	*p*	$\eta_{p}^{2}$
		*M*	*SD*	*M*	*SD*	*M*	*SD*			
Attractiveness		2.46_c_	0.87	4.39_a_	0.70	3.15_b_	1.02	118.83	<0.001	0.87
Hit	%	57.89_b_	21.22	55.92_b_	18.06	69.96_a_	18.84	7.66	0.002	0.30
	Reaction time	1351.3	527.6	1440.0_b_	610.5	1242.1_a_	457.7	7.57	0.002	0.30
	Confidence	2.38_a_	0.32	2.21_b_	0.40	2.29	0.33	4.08	0.025	0.19
Correct rejection	%	72.15_a_	19.59	69.96_a_	19.13	45.18_b_	20.74	24.25	<0.001	0.57
	Reaction time	1322.4_a_	393.5	1347.4	418.2	1447.3_b_	505.2	4.24	0.022	0.19
	Confidence	2.18	0.46	2.21_a_	0.41	2.01_b_	0.46	4.44	0.019	0.20
Area under the curve (AUC)		0.73_a_	0.12	0.68_a_	0.12	0.61_b_	0.10	8.81	0.001	0.33
Discrimination accuracy (Pr)		0.28_a_	0.23	0.24	0.19	0.14_b_	0.19	4.11	0.025	0.19
Response bias (Br)		0.40_a_	0.22	0.41_a_	0.20	0.63_b_	0.20	17.63	<0.001	0.50

*Means in the same row that do not share subscripts differ at p < 0.05 according to the Bonferroni procedure. Reaction time: geometric average response times (in ms) after logarithmic conversion. Attractiveness ratings were made on a 7-point scale (1: extremely unattractive, 2: very unattractive, 3: moderately unattractive, 4: neither, 5: moderately attractive, 6: very attractive, 7: extremely attractive). Confidence ratings were made on a 3-point scale (1: not at all confident, 2: somewhat confident, 3: extremely confident).*

**FIGURE 3:**
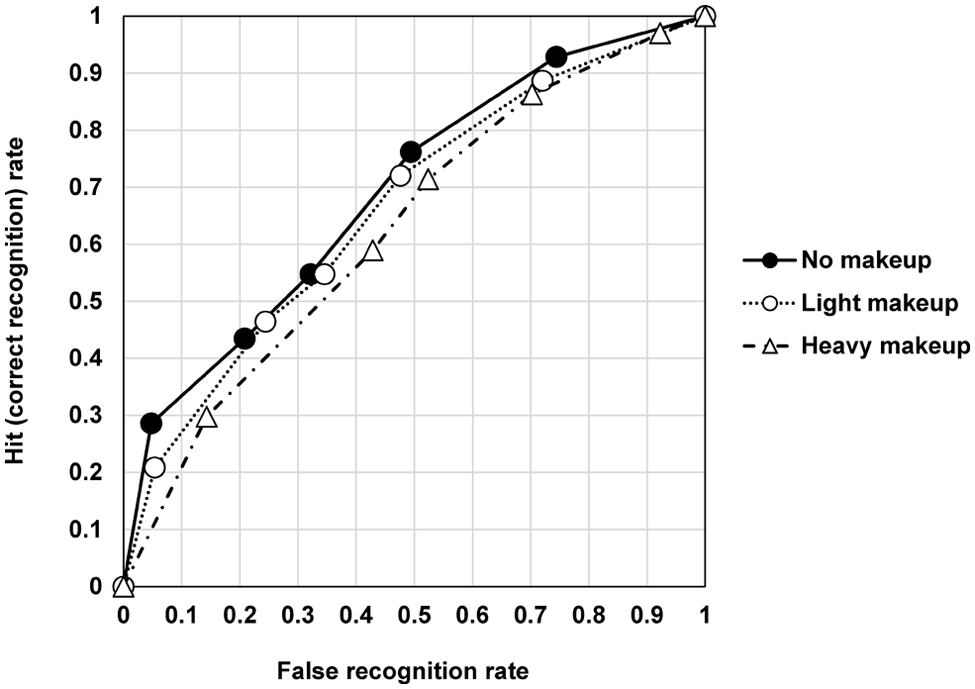
**Receiver operating characteristic (ROC) curves of no makeup, light makeup, and heavy makeup based on each average**.

For the recognition task, hit and correct rejection rates differed significantly between the categories: the hit rate was higher and the correct rejection rate was lower for heavy makeup than for no makeup and light makeup. **Figure 3** shows the receiver operating characteristic curves of the no makeup, light makeup, and heavy makeup faces. The AUC was significantly smaller for heavy makeup faces than for no makeup and light makeup faces. There was a significant linear effect across conditions that suggests better recognition for a lesser amount of makeup, *F*(1,37) = 17.83, *p* < 0.001, $\eta_{p}^{2}=0.33$. Similarly, discrimination accuracy (*Pr*) was lowest for heavy makeup faces. Again, a linear effect across conditions was significant, *F*(1,37) = 8.18, *p* = 0.007, $\eta_{p}^{2}=0.18$. Therefore, the high hit rate for heavy makeup faces is due to a liberal response criterion. This observation was confirmed statistically: the response bias (*Br*) differed significantly across the categories, *F*(2,36) = 17.63, *p* < 0.001, $\eta_{p}^{2}=0.50$. *Post hoc* pair-wise comparisons showed that *Br* was higher for heavy makeup faces than for no makeup and light makeup faces, *p*s < 0.05. Moreover, heavy makeup faces were different from the other faces in terms of the reaction time and confidence rating data. Heavy makeup faces were associated with the shortest correct recognition (hit) time and the longest correct rejection time. Correct recognition time and correct rejection time differed significantly across the categories, *F*(2,36) = 7.57, *p* = 0.002, $\eta_{p}^{2}=0.30$ and *F*(2,36) = 4.24, *p* = 0.022, $\eta_{p}^{2}=0.19$, respectively. *Post hoc* comparisons showed that correct recognition was faster for heavy makeup than for light makeup and that correct rejection was faster for heavy makeup than for no makeup, *p*s < 0.05. Confidence in correct rejection also differed significantly across the categories, *F*(2,36) = 4.44, *p* = 0.019, $\eta_{p}^{2}=0.20$. *Post hoc* comparisons showed that heavy makeup faces were judged with lower confidence as compared to light make up faces, *p* < 0.05.

In the impression reporting session at the end of the experiment, both light and heavy makeup faces were chosen more frequently than no makeup faces as faces that made an impression. The rate of choice was 27.4% for no makeup, 34.7% for light makeup, and 37.9% for heavy makeup. The reasons for choosing these faces differed across makeup categories: asymmetry, close-set eyes, acne, and moles for no makeup faces; beautiful, gentle-looking, and feminine for light makeup faces; and thin, haughty, and unfavorable for heavy makeup faces.

## Discussion

The present study examined the effects of light and heavy makeup on the rating of attractiveness and face recognition. The results showed that facial attractiveness was judged greatest for faces with light makeup. Heavy makeup was in second place, and no makeup received the lowest ratings. The accuracy of facial recognition was higher for faces without makeup than for faces with light or heavy makeup. Therefore, facial distinctiveness was shown to be more associated with facial recognition than with facial attractiveness. In addition, the response bias for heavy makeup faces was significantly more liberal compared with no makeup and light makeup faces. That is, faces with heavy makeup were often recognized falsely. In the impression reporting session at the end of the experiment, the participants replied that the heavy makeup stood out in their memory and it made as much of an impression as light makeup. Nevertheless, the results showed that facial recognition was in fact lower for the heavily made-up faces than for the other faces.

Attractiveness was rated higher for faces with makeup than for faces without makeup. Makeup, whether light or heavy, creates a positive impression on observers by concealing and minimalizing the negative elements of one’s natural face. This is often a primary reason why women wear makeup on a daily basis. For instance, facial features that are commonly considered negative (by societal norms) include asymmetric features, the shape of one’s eyes or lips, and uneven skin tone. The texture of facial skin affects the perception of a subject’s sex (Kloth et al., 2015). Aging has a negative effect on the skin condition that is important for aesthetic evaluation of the face. Image analysis of melanin and hemoglobin has revealed that aging increases skin color heterogeneity (Kikuchi et al., 2015). A three-dimensional (3D) skin surface micro-topography has shown that the pores, ridges, and furrows of the skin surface are deteriorated with aging (Masuda et al., 2014). Makeup can modify the distinctiveness of facial features by arranging color, texture, and shapes. As a result, wearing makeup enhances facial attractiveness (Mulhern et al., 2003). It has been reported that attractiveness ratings are negatively correlated with distinctiveness ratings (Rhodes and Tremewan, 1996; Wickham and Morris, 2003; Peskin and Newell, 2004). Makeup reduces facial distinctiveness and might therefore work to approximate a more average face. Moreover, another study suggests that an increase in luminance contrast between facial features and facial skin enhances the attractiveness of female faces (Russell, 2003, 2009); particularly, lip redness has been reported to enhance apparent femininity in Caucasian faces (Stephen and McKeegan, 2010). Thus, the averageness of a face, luminance contrast and femininity are important elements in judging an attractive female face.

In this study, faces with light makeup were judged as more attractive than those with heavy makeup. In a previous study (Etcoff et al., 2011), faces with natural makeup were ranked higher than those with no makeup in judgments of likability and trustworthiness, suggesting that natural makeup also impacts a sense of social cooperation as well as attractiveness and competence. The same study showed that judgments for faces with heavily contrasting (glamorous) makeup led to judgments similar to those of natural makeup faces when viewing times were brief (250 ms). However, with longer (unlimited) viewing times, the ratings of likability and trustworthiness for the faces with heavy makeup dropped down, while these faces still elicited higher ratings of attractiveness and competence than did no makeup faces. In contrast, faces with light makeup were rated as more attractive, competent, likable, and trustworthy than faces with no makeup regardless of viewing duration. Differences in the evaluation of such social attitudes may be related to corresponding differences in evaluations of the attractiveness of faces with light and heavy makeup in the present study. For example, lighter makeup may be more likely to convey trustworthiness than heavier makeup. The current finding that light makeup received more positive evaluations than heavy makeup is consistent with this speculation. Although this may be due to the effect of recent fashion trends in Japan, it is also possible that faces with lighter makeup, which presumably allow more personality to be seen, may be evaluated more positively. In connection with this finding, it is noteworthy that women tend to wear heavier makeup than that is preferred by others (Jones et al., 2014). This tendency is argued to be because women have an inaccurate perception of others’ preferences about cosmetics. Taken together with the present study, the finding that lighter makeup is preferred by others may be a useful hint for everyone who wears makeup.

The accuracy of face recognition was higher for faces with no makeup and light makeup than for faces with heavy makeup. Natural faces have idiosyncratic features. For example, Schulz et al. (2012) reported that compared with caricatured faces in which metric differences between each individual face and a gender-matched average face were selectively exaggerated, natural faces exhibited a larger learning advantage when these stimuli were matched in perceived distinctiveness. Participants’ reports in the present study also suggest that distinctiveness for faces without makeup may be due to idiosyncratic facial morphology and skin surface features. Therefore, it is reasonable that faces without makeup are better recognized, because the absence of makeup highlights individual characteristics. The same inference can be applied to the advantage of light makeup over heavy makeup. Light makeup is a natural application of cosmetics that takes advantage of an individual’s face. Therefore, the distinctiveness of facial features in light makeup faces was more retained relative to faces with heavy makeup. The superiority of light makeup over heavy makeup in facial recognition is consistent with the findings of Ueda and Koyama (2010), who used a very short retention period. However, they reported that faces with light makeup were better recognized than faces with no makeup, which disagrees with the present finding of better recognition for a lesser amount of makeup. This inconsistency may be due to the differences in retention duration (150 ms vs. several minutes) or in the characteristics of the light makeup. Because they did not report the exact features of the light makeup they used, direct comparison with the present finding is difficult. If they used a type of makeup that emphasized idiosyncratic facial features, it is understandable that recognition performance was better for light makeup than for no makeup.

Interestingly, poor memory performance for heavy makeup faces was associated with a liberal response bias. Novel faces with heavy makeup were often recognized falsely as if they were faces the observer had previously seen. This is possibly because such makeup obscures individual facial features, and the distinctiveness of the makeup itself becomes prominent. That is, participants had a strong memory for the style of makeup but they were not able to identify individual faces. This effect of heavy makeup may lead to an interesting speculation. In the past, working women in Japan often wore heavy makeup. Before the enactment of laws protecting women in the workplace, women were not a major presence in the workforce. Heavy makeup may have been an effort to increase the recognition of women in work places. In a sense, it was effective. However, at the same time, heavy makeup has an untoward effect: it obscures individual characteristics. Currently, women have essential roles in many work places. Therefore, light makeup may become more popular and more effective because it facilitates individual recognition. Of course, depending on circumstances and purpose, heavy makeup may be more effective as an element of interpersonal communication than light makeup; in some situations, heavier makeup may even be considered more attractive.

Applying facial cosmetics affects women’s self-images positively (Cash et al., 1989). The results of the present study suggest that light makeup accentuates individual attractiveness while heavy makeup emphasizes the attractiveness of the makeup itself. Therefore, wearing light makeup may be more effective in promoting a positive self-image as compared to wearing heavy makeup that obscures individual characteristics. The use of cosmetics has been suggested to be a tool for self-presentation and social impression management (Guthrie et al., 2008). It is worth examining how light makeup and heavy makeup influence interpersonal cognition such as trustworthiness and competence (Hosoda et al., 2003; Etcoff et al., 2011). Moreover, the present findings may contribute to further technological advances in facial coding software (Lewinski, 2015) and makeup recommendation systems (Scherbaum et al., 2011; Chung, 2014). For example, the eye line, cheeks, and lip modified by makeup may affect the tracking points of the face. Heavy makeup may produce more bias than light makeup in facial coding.

There are several limitations in this study. First, the faces featuring no makeup, light makeup, and heavy makeup were of different individuals. Therefore, the distinctiveness of individual faces might affect recognition performance. It is very costly to prepare three types of makeup for each individual model while maintaining a high standard of the quality of photographs. However, the effect of makeup on a single individual face needs to be tested in a future study. Second, facial makeup images used in the present study were digitally retouched. Although the quality of the final images was carefully controlled by a professional makeup artist, actual makeup and digital manipulation might have different effects. This issue will be resolved by replicating this study using models with actual makeup alone. Third, only female participants were recruited in this study. In connection to the behavior of partner selection, it is worth examining whether men show a similar pattern of results.

The present study assessed the effects of light and heavy makeup in determining perceived facial attractiveness and face recognition using an old/new task. The results showed that the highest ratings of attractiveness were for faces with light makeup; in addition, recognition accuracy was highest for faces with no makeup and lowest for faces with heavy makeup. These results show that the distinctiveness of a face, rather than its attractiveness, has a greater effect on recognition accuracy. Moreover, it was found that people tended to mistake unseen faces with heavy makeup for previously presented ones. The present study suggests that, at least under current fashion trends, light makeup is preferable to heavy makeup because it is viewed as more attractive and allows for greater expression of individual personality and easier recognition by others.

## Author Contributions

KT and HN planned, collected, analyzed, interpreted the data, and wrote the paper. HO contributed for the preparation of stimulus.

## Conflict of Interest Statement

KT and HO are employed by Shiseido Global Innovation Center, Shiseido Co., Ltd., a company which manufactures cosmetics, and HN serves as a technical advisor for Shiseido Co., Ltd. There are no patents, products in development or marketed products to declare.
